# Potential impacts of aquatic pollutants: sub-clinical antibiotic concentrations induce genome changes and promote antibiotic resistance

**DOI:** 10.3389/fmicb.2015.00803

**Published:** 2015-08-05

**Authors:** Louise Chow, Liette Waldron, Michael R. Gillings

**Affiliations:** Emma Veritas Laboratory, Department of Biological Sciences, Macquarie UniversitySydney, NSW, Australia

**Keywords:** antibiotic resistance, microbiome, antibiotic pollution, SOS response, evolution

## Abstract

Antibiotics are disseminated into aquatic environments via human waste streams and agricultural run-off. Here they can persist at low, but biologically relevant, concentrations. Antibiotic pollution establishes a selection gradient for resistance and may also raise the frequency of events that generate resistance: point mutations; recombination; and lateral gene transfer. This study examined the response of bacteria to sub-inhibitory levels of antibiotics. *Pseudomonas aeruginosa* and *Pseudomonas protegens* were exposed kanamycin, tetracycline or ciprofloxacin at 1/10 the minimal inhibitory concentration (MIC) in a serial streaking experiment over 40 passages. Significant changes in rep-PCR fingerprints were noted in both species when exposed to sub-inhibitory antibiotic concentrations. These changes were observed in as few as five passages, despite the fact that the protocols used sample less than 0.3% of the genome, in turn suggesting much more widespread alterations to sequence and genome architecture. Experimental lines also displayed variant colony morphologies. The final MICs were significantly higher in some experimental lineages of *P. protegens*, suggesting that 1/10 the MIC induces *de-novo* mutation events that generate resistance phenotypes. The implications of these results are clear: exposure of the environmental microbiome to antibiotic pollution will induce similar changes, including generating newly resistant species that may be of significant concern for human health.

## Introduction

Antibiotic resistance has been identified as one of the greatest threats to human health for the twenty-first century by the World Health Organisation (WHO, 2014). Overuse and misuse of antibiotics in the medical and agricultural sectors have contributed to the problem, and it is estimated that 70% of pathogens now exhibit resistance to at least one or more antibiotics (Berdy, 2012). In most cases the risk of death is doubled if the individual is infected with a resistant strain of bacteria. In the United States in 2013, there were 23,000 confirmed deaths due to Antibiotic resistance (US CDC) and Europe reports 25,000 deaths per year (2007, ECDC).

The primary human use of antibiotics is medicinal, where they are used to treat a range of bacterial infections. However, misuse and overuse of antibiotics are contributing to the development of antibacterial resistance. Incorrect prescription of antibiotics, unnecessarily high dosages and over-use all promote resistance (Campoccia et al., 2010; Andersson and Hughes, 2012; Hvistendahl, 2012; Witte, 2013). Antibiotics are also extensively used in agriculture and aquaculture to prevent disease and as a growth promoter (Hilbert and Smulders, 2004; Bednorz et al., 2013). It has been estimated that 50–70% of antibiotics produced in the United States of America are used in agriculture (Lipsitch et al., 2002; Berge et al., 2005).

A relatively small amount of the antibiotics consumed by humans and animals are actually absorbed, with some 30–90% of antibiotics excreted unchanged and released into waste treatment facilities or directly into the environment (Sarmah et al., 2006). Antibiotics, along with heavy metals, disinfectants and genes conferring resistance are disseminated into the environment via human waste streams, agricultural run-off (Su et al., 2014) and effluent from antibiotic production factories (Li et al., 2009, 2010). Current waste treatment methods are often unable to remove these substances, and the water is either reclaimed (Wang et al., 2014) or released into the environment via rivers (Pruden et al., 2006; Storteboom et al., 2010), estuaries or the ocean (Lapara et al., 2011; Wang et al., 2014). The release of these substances into the environment should be thought of as a significant component of soil and water pollution.

Waste water treatment facilities and aquatic environments can become hotspots for the generation and acquisition of resistance. The presence of selective agents such as antibiotics, heavy metals and disinfectants, combined with genes conferring resistance, mobile elements such as transposons, plasmids, and integrons, and diverse microorganisms creates an ideal environment to generate resistance through mutation or lateral gene transfer.

Many studies have investigated the effect of clinical, or inhibitory levels, of antibiotics on the generation of antibiotic resistance. However, there is increasing evidence that sub-inhibitory levels of antibiotics may have significant effects on bacterial populations. A gradient of antibiotic concentration forms around human activities. Within the human microbiome there may be a gradient along the digestive tract, while dissemination of antibiotics via waste water will generate a gradient of antibiotic concentration spreading outwards from human population centers.

Sub-inhibitory levels of antibiotics are known to trigger the SOS response, a broad response to DNA damage that has been documented in many bacterial species. It may play a significant role in the generation of antibiotic resistance, as it can increase the rates of mutation and lateral gene transfer (Baharoglu and Mazel, 2014). It is triggered by the occurrence of single stranded DNA resulting from DNA damage, or inhibition of the processes involved in DNA replication. The SOS response is mediated by the LexA repressor. Under normal conditions, LexA prevents SOS genes from being expressed. Under stressful conditions, the protein RecA is recruited onto single stranded DNA where it stimulates cleavage of the LexA repressor, inactivating it and therefore allowing the expression of approximately 40 SOS genes. SOS genes are often involved in DNA repair (Laureti et al., 2013; Baharoglu and Mazel, 2014).

It is well documented that lethal concentrations of antibiotics can induce the SOS response in bacteria (Miller et al., 2004; Michel, 2005). It has also been suggested that sub-inhibitory levels of antibiotics, as those discussed above, may be more relevant to the problem of antibiotic resistance than lethal concentrations of antibiotics (Andersson and Hughes, 2012; Hughes and Andersson, 2012; Laureti et al., 2013). Lethal concentrations exert a strong selective pressure on bacteria, whereby they either die or they acquire mutations allowing them to survive. When exposed to sub-inhibitory levels of antibiotics, most bacteria survive with little effect on growth, and the SOS response is initiated. This, in turn, increases general rates of mutation and lateral gene transfer amongst all bacteria in a population, adding to any extant diversity upon which natural selection can operate. It is also thought that humans may be inadvertently selecting for lineages of bacteria with a greater ability to evolve through increased basal rates of mutation and lateral gene transfer (Gillings and Stokes, 2012).

Sub-inhibitory concentrations of antibiotics polluting areas surrounding human activity may be affecting: (i) the rates at which bacteria can generate variation; and (ii) the rates at which advantageous mutations fix in natural environments. However, there has been little or no empirical testing of these ideas.

In this study, two species of *Pseudomonas* were passaged as single colony transfers on media containing 1/10 their respective minimum inhibitory concentrations for three different classes of antibiotics. This experiment was designed to test the genotypic and phenotypic effects of realistic levels of antibiotic pollution.

## Materials and methods

### Bacterial isolates

Isolates of two species were selected for this study: *Pseudomonas aeruginosa* strain PA14; and *P. protegens* strain PF-5. These species were chosen as they encompass both clinical and environmental representatives of the genus. Both strains have been genome sequenced (GenBank: AY273869.1 GenBank: CP000076.1, He et al., 2004; Paulsen et al., 2005). *P. aeruginosa* PA14 is an opportunistic bacterium that causes infections in hospitals and cystic fibrosis patients. *P. protegens* PF-5 (formerly *Pseudomonas fluorescens* PF-5) is a common soil bacterium studied for its potential biocontrol properties (Loper et al., 2012).

*P. aeruginosa* PA14 was obtained from Professor Joyce Loper, Oregon State University and *P. protegens* PF-5 was obtained from Professor Ian Paulsen, Macquarie University. Bacteria were maintained on LB Agar plates (0.01% tryptone, 0.005% yeast extract, 0.005% sodium chloride, 0.015% Agar) at 25°C. A second isolate of *P. protegens* PF-5 was obtained that had been routinely maintained of 100 μg/ml ampicillin, which is a common laboratory practice. This isolate was examined to determine whether maintenance on ampicillin affects the resistance of *P. protegens* PF-5, and will be referred to as *P. protegens* PF-5A. Single colonies were re-suspended in equal parts 30% glycerol and M9 salts and held at -80°C for long term storage.

### Antibiotic treatments

Three antibiotics were selected for this study, each with different modes of action: kanamycin; tetracycline; and ciprofloxacin. Kanamycin is an aminoglycoside antibiotic which binds to the 30S ribosomal subunit and inhibits prevents protein synthesis (Misumi and Tanaka, 1980). Tetracycline is a polyketide antibiotic that is similar to kanamycin in that it binds to the 30S ribosomal subunit, however it prevents aminoacyl-tRNAs attaching to the ribosome, which in turn prevents addition of amino acids to growing polypeptide chains (Chopra and Roberts, 2001). Ciprofloxacin is a second generation fluoroquinolone used to treat a broad spectrum of infections. It inhibits DNA gyrase, which in turn prevents DNA replication (Lebel, 1988).

### Determination of minimum inhibitory concentration

The minimum inhibitory concentration (MIC) was determined for each isolate against the three antibiotics following established methodology (Wiegand et al., 2008). MICs were determined in microtitre trays containing a serial dilution of the relevant antibiotic in Luria-Bertani medium (0.01% tryptone, 0.005% yeast extract, 0.005% sodium chloride). Wells were inoculated with bacteria prepared from an overnight culture and diluted to an optical density of 0.01. The concentration of antibiotic in test wells ranged from 32 to 0.0156 mg/L for ciprofloxacin and 512–0.0156 mg/L for tetracycline and kanamycin. A growth control containing only the suspension of bacteria and a sterility control containing only medium were included on each plate. Plates were incubated at 25°C for 24 h and then the optical density was read on a Pherastar FS spectrometer at 540 nm. Relative optical density was plotted against antibiotic concentration to determine the MICs, which were defined as no visible growth in the wells.

To determine statistical significance of differences in MIC, a One-Way analysis of variance (ANOVA) was performed. Growth data were expressed as the ratio of growth in the presence of antibiotics against growth in the control. This standardized the data prior to the ANOVA.

### DNA extraction

DNA was extracted from bacterial cultures using a bead-beating method (Yeates and Gillings, 1998; Gillings, 2014). Briefly, a single, well isolated colony from an overnight culture was resuspended in a lysing matrix tube with sodium phosphate buffer and MT buffer (MP Biomedicals) or with CLS-TC buffer (MP Biomedicals). Preliminary testing indicated no significant difference between sodium phosphate/MT buffer and CLS-TC buffer, therefore CLS-TC buffer was used for the remainder of the study as it was more economical. Cells were physically lysed by treatment in a FastPrep FP120 (BIO 101 Savant) machine for 30 s at 5.5 m/s before being centrifuged in an Eppendorf 5417C, for 5 min at 14,000 g. Protein precipitation, binding, washing and subsequent elution of DNA in TE buffer were as previously described (Yeates and Gillings, 1998; Gillings, 2014). Purified DNA was stored at −20°C.

### Repetitive element PCR

DNA fingerprints were generated using ERIC-PCR, REP-PCR or BOX-PCR (Versalovic et al., 1991; Martin et al., 1992) with the modifications previously outlined (Gillings and Holley, 1997). One μL of DNA was mixed with 9 μL of Genereleaser™ (Bioventures Inc.) in a 0.5 mL PCR strip tube, and heated on high for 7 min in a 650 W microwave oven with a microwave sink. Tubes were then held at 80°C for 5 min in an Eppendorf Master Cycle Epigradient S PCR machine, before 40 μL of PCR master mix was mixed into each tube. The PCR master mix per reaction was as follows: 11 μL PCR water, 25 μL GoTaq® white (Promega), 2.5 μL 25 mM MgCl_2_, 0.5 μL 1 mg/ml RNAse, 1 μL 50 μM of the relevant rep-PCR primer. Negative controls containing Genereleaser™ only and water only were included in each PCR. The appropriate PCR cycle was then performed (Table 1). BOX, ERIC, and REP primers were synthesized by Sigma-Aldrich Inc.

**Table 1:** **Thermal cycling programs and primers used to generate DNA fingerprints using Rep-PCR**.

**Rep PCR**	**Primers**	**Thermal cycle**
BOX	BOXA1R: 5′CTACGGCAAGGCGACGCTGACG	94°C 3 min 94°C 30 s × 35 52°C 30 s × 35 68°C 8 min × 35 68°C 15 min × 35 4°C hold
ERIC	ERIC1R: 5′ATGTAAGCTCCTGGGGATTCAC ERIC 2: 5′AAGTAAGTGACTGGGGTGAGCG	94°C 3 min 94°C 30 s × 35 52°C 30 s × 35 68°C 8 min × 35 68°C 15 min × 35 4°C hold
REP	REPR: 5′TTCGCYGGCAAGCCRGCTCC REP F: 5′GGCTTGCCRGCGAARRGGCC	94°C 3 min 94°C 30 s × 35 65°C 30 s × 35 72°C 8 min × 35 72°C 15 min × 35 4°C hold

### Agarose electrophoresis

PCR products were separated on 2% agarose gels poured in Tris-Borate-EDTA (TBE) buffer (Russell and Sambrook, 2001). DNA samples were loaded with one quarter volume of bromophenol blue loading dye (0.45 M Tris-borate, 0.01 EDTA, 40% sucrose, 0.25% bromophenol blue). A 100 base pair ladder (Crown Scientific) was included on each gel. Gels were run in TBE at 110 v for 50–80 min. Gels were stained with GelRed™ (Biotium) and DNA visualized under UV light. Gel images were captured using a Gel logic 2200 PRO camera and Carestream MI computer software.

### Serial plating experiments

A single, well isolated colony of each species was chosen to (as far as possible) eliminate any extant variation amongst cells. This single colony was then used to inoculate the control LB agar plates; LB plates containing 1/10 the MIC for kanamycin; LB plates containing 1/10 the MIC for tetracycline; and LB plates containing 1/10 the MIC for ciprofloxacin, each in triplicate (Table 2). Plates were incubated at 25°C for 48 h, referred to here, for convenience, as one generation.

**Table 2:** **Concentrations of antibiotics used in serial plating experiments, corresponding to 1/10 the experimentally determined MIC**.

**Antibiotics**	**Bacterial isolates**		
	**PA14 (mg/L)**	**PF5 (mg/L)**	**PF5A (mg/L)**
Kanamycin	25.6	0.8	0.8
Tetracycline	25.6	25.6	25.6
Ciprofloxacin	0.0125	0.025	0.025

After incubation for 48 h, a single well-separated colony from each plate was used to continue the serial plating. After five generations, three single colonies were randomly selected from each plate for DNA extraction and PCR analysis, the first of these three would also be used to continue the serial plating. Repetitive Element PCRs were carried out to monitor changes in DNA patterns and to monitor for possible contamination of the cultures.

### DNA banding analysis

Images captured of the gels were analyzed to identify changes in the banding patterns, indicative of changes in the genome of the sample. Changes were scored against a control profile to calculate the similarity coefficient (F) using the formula devised by Nei and Li (1979):

$$\begin{array}{l} \text{F}=2{\text{N}}_{\text{xy}}∕\left({\text{N}}_{\text{x}}+{\text{N}}_{\text{y}}\right) \end{array}$$

Where Nx and Ny are the number of bands in lane x and lane y respectively and Nxy is the number of bands that lane x and lane y share. Samples with an *F*-value of 1 are identical while a value of 0 indicates no similarity. Scoring of the bands was carried out blind by an individual not involved in the Rep-PCR process, to remove the possibility of bias. The *F*-values for the various antibiotic treatments were plotted as a scatter graph to illustrate the spectrum of variation.

### Changes in colony morphology

To examine colony morphology at the end of the experiment, colonies of all lines from generation 40 were streaked onto LB agar plates and incubated for 48 h at 25°C. Images of single colonies were captured using a Motic BA300 compound microscope with a 4x lens, mounted with a Moticam 2 2.0MP camera and were analyzed using DigiLabII-C and Motic Images Plus 2.0 computer programs.

## Results

### Colony morphology changes

**Figure 1:**
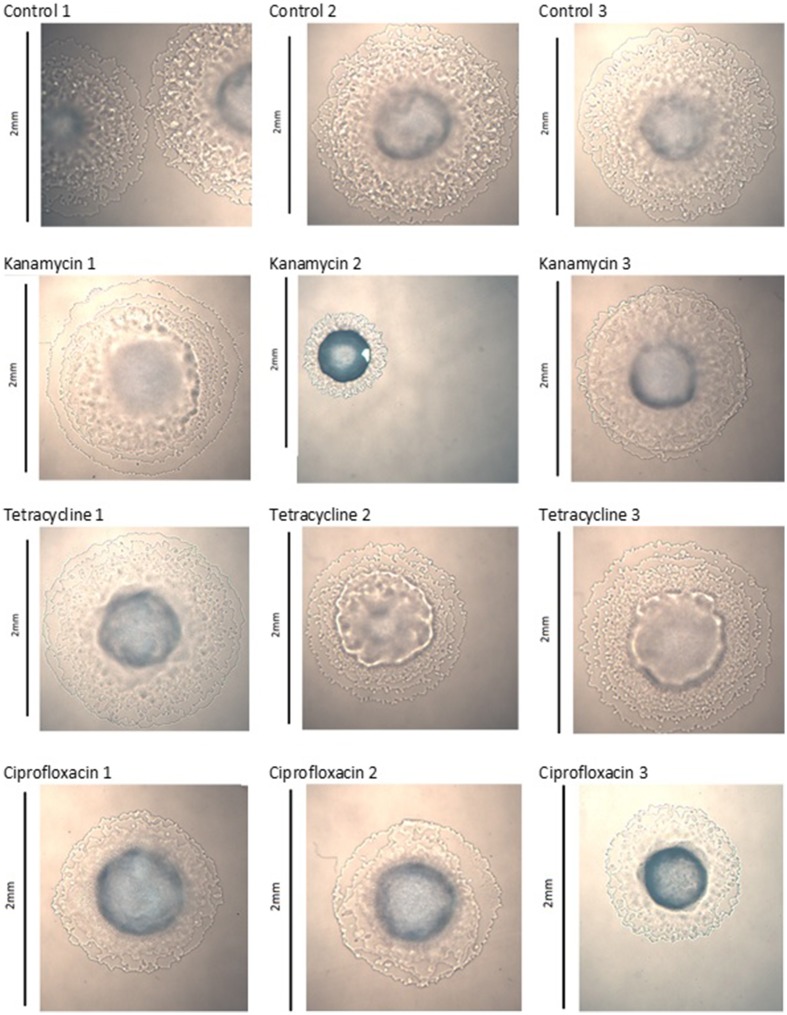
*****Pseudomonas aeruginosa*** PA14 colony morphology at generation 40**.

Images captured of colonies at generation 40 show significant morphological changes between treatment groups. The three control lines of *P. aeruginosa* PA14 displayed no significant changes, kanamycin line 2, tetracycline lines 2 and 3, and ciprofloxacin line 3 exhibited significant changes to their colony morphology (Figure 1). The three control lines of *P. protegens* PF-5 displayed no significant changes, kanamycin line 3 and tetracycline line 3 exhibited significant morphological differences. The three ciprofloxacin lines were relatively unchanged (Figure S1). The three control lines and three tetracycline lines of *P. protegens* PF-5A had similar colonies. All three kanamycin lines had significantly changed colonies, as had lines 2 and 3 of the ciprofloxacin treatment (Figure S2).

### Detectable genome changes

**Figure 2:**
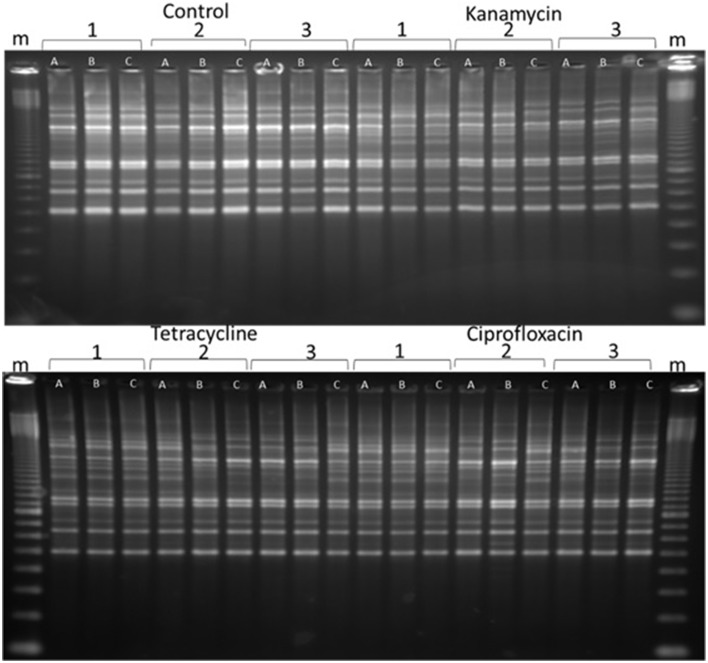
**A representative sample of BOX-PCR products**. BOX-PCR was performed on generation 40 *Pseudomonas protegens*. Lanes are labeled as follows: *m* = 100 bp ladder. Antibiotic treatments are noted as independent lines within each treatment (1, 2, or 3). Three colonies were tested from each line. For further examples see Supplementary Material (Figures S3–S6).

BOX, ERIC, and REP-PCRs were carried out to detect genome changes. The basis of these PCRs is explained in Gillings and Holley (1997), but, in brief, relies on amplification of regions between two random, but reproducible priming sites. Consequently, amplicons are sensitive to both mutations in the priming sites and indels across the amplified regions. After testing both species with ERIC, REP and BOX primers, BOX-PCR was determined as the best method to examine changes. BOX-PCRs were conducted on triplicates of all lines every five generations. Experimental lines often exhibited changes in banding patterns, while the control lines remained the same, indicating that the changes were due to exposure to 1/10 MIC antibiotics (Figure 2). Changes were apparent after as few as five passages (evidence not presented), and increased in frequency as the experiment progressed, until they were present in the majority of experimental lines after 40 passages (Figure 2, Figures S3–S6).

Two features were notable in the lines exposed to sub-inhibitory antibiotic concentrations. In general, polymorphisms were commonly exhibited in experimental lineages, and often, replicates from single lineages exhibited diversity, demonstrating an ongoing instability within each generation. Further, similar changes to banding patterns were often observed in independent lineages, suggesting that similar events (such as transpositions or prophage activation) were being promoted within independent lines by the antibiotic treatment (Figure 2).

**Figure 3:**
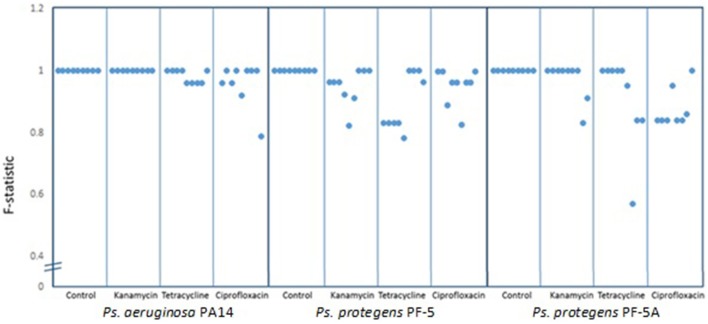
**Similarity co-efficient (F) of BOX patterns from experimental lines at generation 40 [F = 2Nxy/(Nx + Ny)] compared to control lines**. Dots represent the averge F-statistic of three samples from each line and each box contains nine lines for each treatment. Scores below 1.0 indicate consistent polymorphisms in BOX-PCR patterns.

To determine the degree of polymorphism amongst the individual experiments, F statistics were calculated. A scatter plot of the F-statistics shows that control lines maintained a uniform BOX-PCR pattern across all three bacterial isolates (PA14, PF-5, and PF-5A) for the 40 generations of the experiment (Figure 3). Amongst the lineages treated with sub-inhibitory antibiotic concentrations, only the kanamycin treatment of PA14 maintained a stable BOX-PCR pattern. All other treatments generated polymorphic banding patterns in at least some of the replicates. The approximate degree to which polymorphisms were generated was in the order of Kan < Tet < Cipro (Figure 3).

### Changes in the MIC

**Figure 4:**
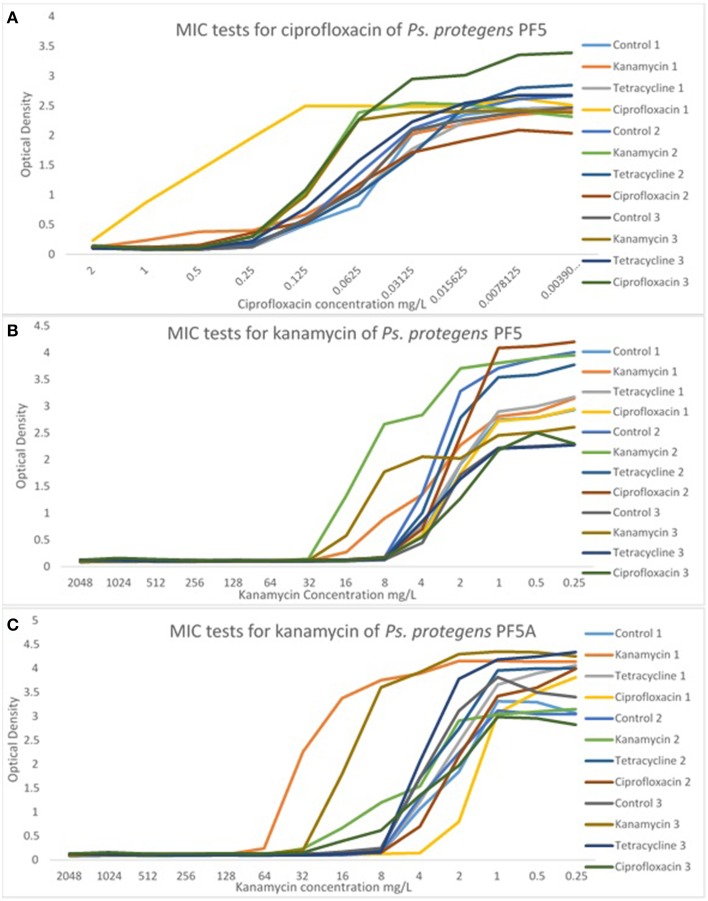
**Representative graphs displaying the MIC of selected antibiotics for the experimental and control lines**. MIC tests for *Ps. protegens* strains are shown for PF5/ciprofloxacin **(A)**, PF5/kanamycin **(B)**, and PF5A/kanamycin **(C)**. For more examples see Supplementary Material (Figures S7–S9).

The MIC of each line was determined at passage 40 in order to detect any significant differences in MICs from the control line and from the starting MIC. There were no significant differences in the MIC of *P. aeruginosa* PA14 for any of the treatment lines. In contrast, there were some significant differences in the MIC of *P. protegens* PF-5 and *P. protegens* PF-5A. A representative sample of MIC graphs are displayed in Figure 4. Figure 4A shows the MIC for ciprofloxacin for all control and experimental lines of *P. protegens* PF5. One line of *P. protegens* PF5 that had been exposed to 1/10 the MIC of ciprofloxacin over the serial plating experiment exhibited a 10-fold increase in MIC for ciprofloxacin (*DF* = 11, *F* = 11.94, *P* < 0.0001). A similar phenomenon was seen in *P. protegens* PF5 (Figure 4B) and *P. protegens* PF5A (Figure 4C) when tested on kanamycin. All six lines that had been exposed to kanamycin over the serial plating experiment had four to eight fold increases in their MIC for kanamycin (*DF* = 11, *F* = 1.96, *P* > 0.05 and *DF* = 11, *F* = 46.04, *P* < 0.0001 respectively). Similar tests conducted on a subset of the kanamycin treated lines at passage 20 did not detect any elevation in MIC.

## Discussion and conclusions

The role of antibiotics as environmental pollutants is attracting more attention, as more concern is being raised about their effects at sub-inhibitory concentrations (Gillings and Stokes, 2012; Andersson and Hughes, 2014). Specific issues include their potential effects on environmental microorganisms, and their potential for triggering complex interactions with the environmental resistome, thereby generating new opportunistic pathogens of relevance to human health (Gillings, 2013). Here we set out to test whether sub-inhibitory concentrations of antibiotics affect the genotype and phenotype of representative clinical and environmental pseudomonads.

*P. aeruginosa* and *P. protegens* were serially plated on agar containing 1/10 the experimentally determined MIC for representatives of three antibiotic classes. This antibiotic concentration was chosen because it induces maximum transcriptional activity (Davies et al., 2006). Exposure to 1/10 the MIC for the panel of antibiotics tested had significant genotypic and phenotypic effects.

Effects on the genomes were immediate and readily detectable. Changes to rep-PCR DNA fingerprints could be detected after as few as five serial transfers on sub-inhibitory antibiotic concentrations. This result is even more remarkable, since BOX-PCR is a fairly insensitive measure of genomic variation, although it generates highly reproducible DNA fingerprints. In this series of experiments, the BOX assay sampled between 15 and 20 kb of DNA, amounting to less than 0.3% of the ~7 Mb pseudomonad genome. If the genome changes are similar in the un-sampled portion of genome, sub-inhibitory antibiotic concentrations are having a widespread and significant effect on DNA sequence, genome architecture, or both.

Sub-inhibitory antibiotic concentrations also induced phenotypic changes. After 40 generations of serial transfer, many of the experimental lines exhibited changes in colony morphology. Perhaps of most significance, all six lines of *P. protegens* maintained on 1/10 the MIC for kanamycin showed up to eight-fold elevation in their MICs for kanamycin by 40 generations. Similarly, one line held on 1/10 MIC for ciprofloxacin also showed an elevated ciprofloxacin MIC by 10-fold. Sub-lethal ciprofloxacin exposure has previously been shown to induce resistance in hypermutable strains of *P. aeruginosa* (Jørgensen et al., 2013). Whether the resistance observed in our experiment is also mediated by mutations in *gyrA* or *gyrB* will have to await sequence analysis.

The changes in MIC we observed are not likely to be the result of selection on pre-existing mutations in the single colony we used to initiate each experiment. A suspension of a single, well isolated colony was used as inoculum for both control and experimental lines of the three pseudomonads tested (PA14, PF-5, and PF-5A). All six kanamycin treated lines (three each for the two independent strains of *P. protegens*) showed elevated MICs for kanamycin by the 40th passage. One line from the ciprofloxacin treatments also showed a ten-fold increase in MIC. For these outcomes to have arisen from pre-existing mutants in the generation zero colony, each of the three kanamycin lines for both strains of *P. protegens* must have been the recipient of an appropriate mutant cell, as must have been the ciprofloxacin lineage. All of these putative mutations must have arisen during the growth of the time zero colonies from a single cell, which seems unlikely. Further, testing of a subset of the lines at passage 20 did not detect any increase in MIC in the kanamycin treated, or any other lines. By passage 20, any pre-existent mutant should have gone to fixation. The most parsimonious explanation for our results is that the changes in MIC were due to *de-novo* mutation.

If our findings are generally applicable, it suggests that similar phenotypic and genotypic changes will occur in all environments where antibiotics reach concentrations of 1/10 the MIC, and that these effects will potentially apply to all members of the environmental microbiota.

The effects of sub-inhibitory antibiotic concentrations observed in our experiments might be driven by the bacterial SOS response, which is known to induce processes that increase mutation, transposition and recombination rates (Gillings, 2013; Andersson and Hughes, 2014). Certainly, ciprofloxacin is a potent inducer of the SOS response, and generated the most extreme changes in BOX fingerprints observed in our experiments. Aminoglycosides such as kanamycin also induce the SOS response, but here tetracycline had an even greater effect on genomic architecture as assessed by BOX-PCR. However, there is no evidence that the diversity we observed is entirely due to the SOS response. The advantage of the approach we have taken here is that all mechanisms that generate variation, including the SOS response, and other potentially novel mechanisms, can be captured.

The concentrations of antibiotics used here may represent typical of levels of antibiotic pollution. There is limited knowledge about the concentrations of antibiotics found in the environment, however it is now known that antibiotics can persist in the environment longer than previously thought. The time that an antibiotic can persist in the environment differs depending on the class of antibiotic and the environmental conditions. Closed bottle tests provide a simple way to measure the biodegradability of antibiotics and indicate whether or not the antibiotic will readily degrade. Classes of antibiotics such as the β-lactams, tetracyclines, macrolides, lincosamides, penicillin, aminoglycosides, carbapenems, nitroimidazoles, polyene-antimycotics, quinolones, sulphonamides, and glycopeptides have all been found to persist over a 28 day testing period (Al-Ahmad et al., 1999; Alexy et al., 2004). High temperatures and exposure to UV light can cause degradation of some antibiotics. Fluoroquinolone antibiotics can degrade in sunlight, however they are readily absorbed onto sediments, where they have been documented persisting up to 80 days with less than 1% of degradation (Marengo et al., 1997). It would be convenient if resistant organisms destroyed or inactivated antibiotics, however the mechanisms that usually allow resistance involve mutation of binding sites or efflux pumps, meaning that antibiotics are not physically altered and may persist in the environment (Levy, 2002). Following one application of manure, antibiotics and antibiotic resistance genes can persist in the soil for approximately 6 months, depending on environmental conditions, during which time it could be dangerous to consume products that have had direct contact with the soil (Marti et al., 2014). Given the significant time frame in which antibiotics can persist in the environment it is highly likely that they will exist at concentrations near 1/10 the MIC.

The concentration at which antibiotics may occur in the environment is affected by several factors: substrate, proximity to source of antibiotics, environmental conditions and the antibiotics themselves. Testing of rivers and oceans have detected the presence of antibiotics, most notably sulphonamides and quinolones which were found at high concentrations in a number of environments. Sulphonamides were detected in water (0.86–1563 μg/L) (Hirsch et al., 1999; Luo et al., 2011; Li et al., 2012; Zhang et al., 2013) and quinolones were detected in sediments and plants (65.5–1166 and 8.37–6532 μg/kg, respectively) (Li et al., 2012). The antibiotic concentration of 1/10 the MIC easily falls into the ranges of antibiotic pollution detected in these waterways, indicating that the results of this study are likely to represent the rates of mutation and recombination taking place in environments polluted by antibiotics.

Very small concentrations of common antibiotics can induce significant genotypic and phenotypic changes in bacterial species. Given the huge quantities of antibiotics that are entering the environment, it is likely that this antibiotic pollution is generating antibiotic resistant organisms that may be a source of newly emerging opportunistic pathogens. These may then pose significant threats to human and animal life. Changes need to be made at every level of antibiotic use, by the individual, in medical practice, in pharmaceutical production, government monitoring and waste treatment, otherwise modern medicine is at a risk of facing a post antibiotic era where infections are harder, and in some cases, impossible to treat.

### Conflict of interest statement

The authors declare that the research was conducted in the absence of any commercial or financial relationships that could be construed as a potential conflict of interest.
