# Supplementary figures and images for: Potential impacts of aquatic pollutants: sub-clinical antibiotic concentrations induce genome changes and promote antibiotic resistance
Source: Front Microbiol. 2015 Aug 5;6:803. doi: 10.3389/fmicb.2015.00803 (PMC4525061; doi:10.3389/fmicb.2015.00803)

**Figure S1.** *Pseudomonas protegens* PF-5 colony morphology at generation 40

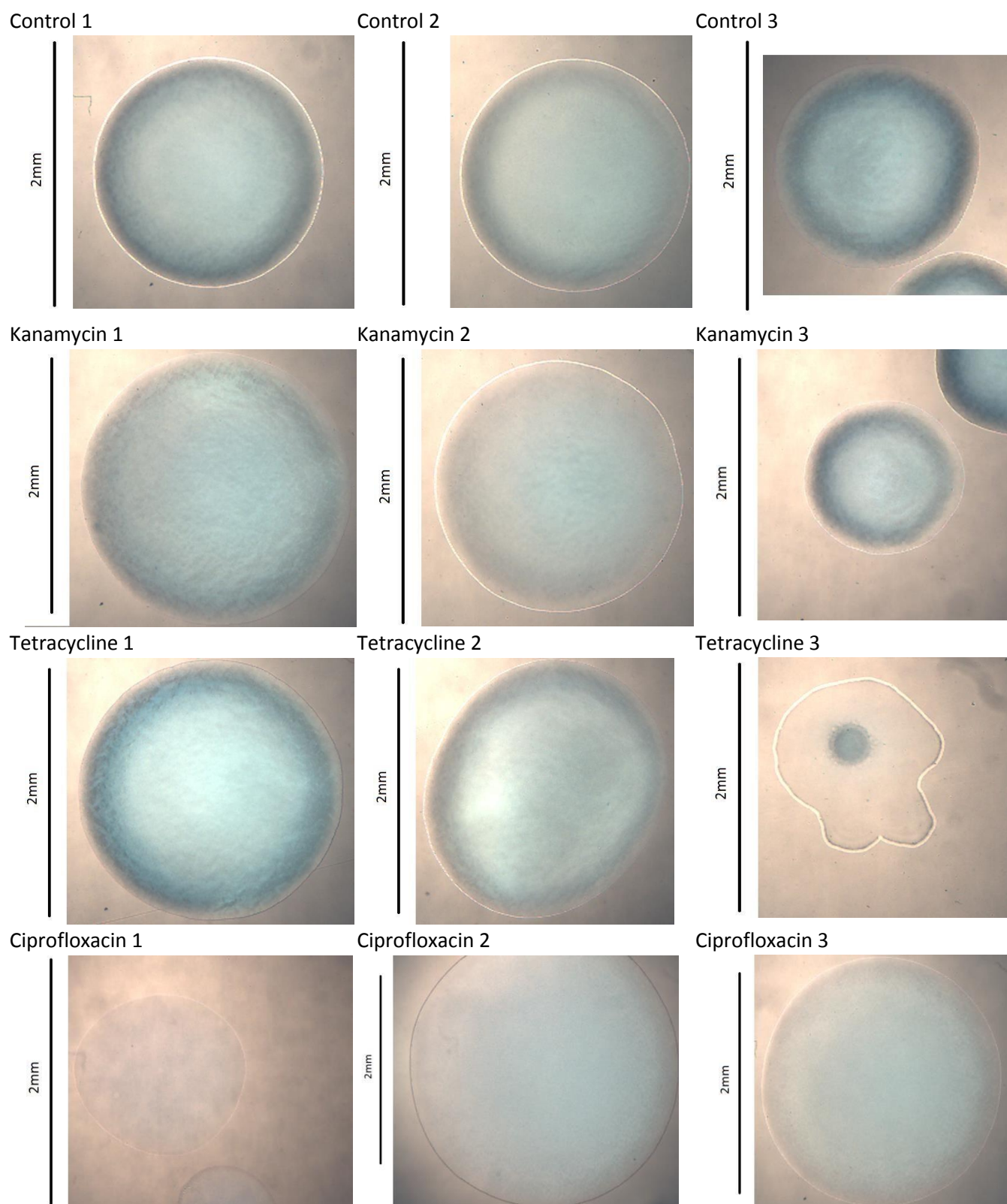

Supplement: Supplementary file 1 [file Image1.PDF]

Figure S3. BOX-PCR of generation 5 *Ps. aeruginosa* PA14. m = 100bp ladder

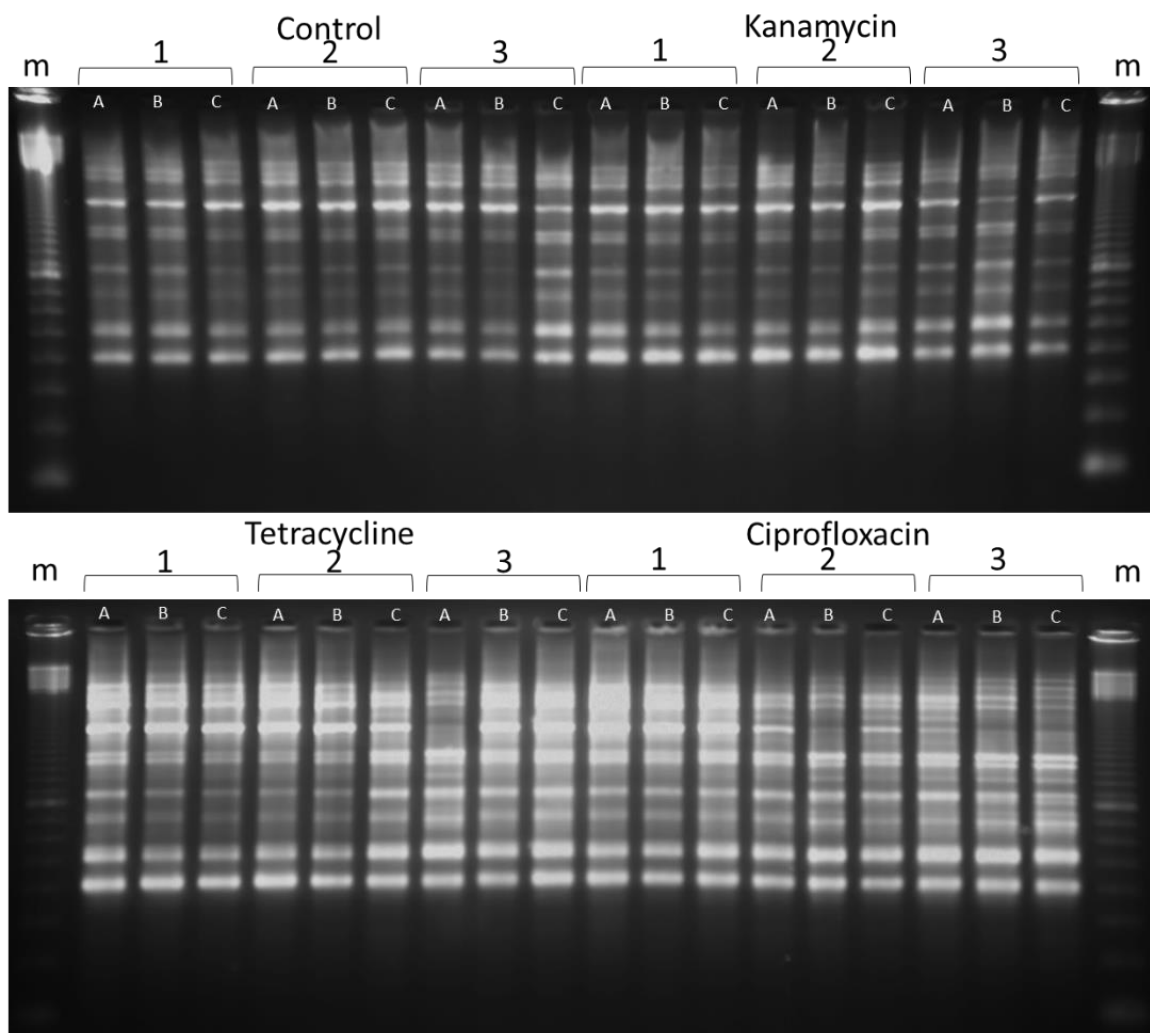

Supplement: Supplementary file 3 [file Image3.PDF]

Figure S4. BOX-PCR of generation 20 *Ps. aeruginosa* PA14. m = 100bp ladder

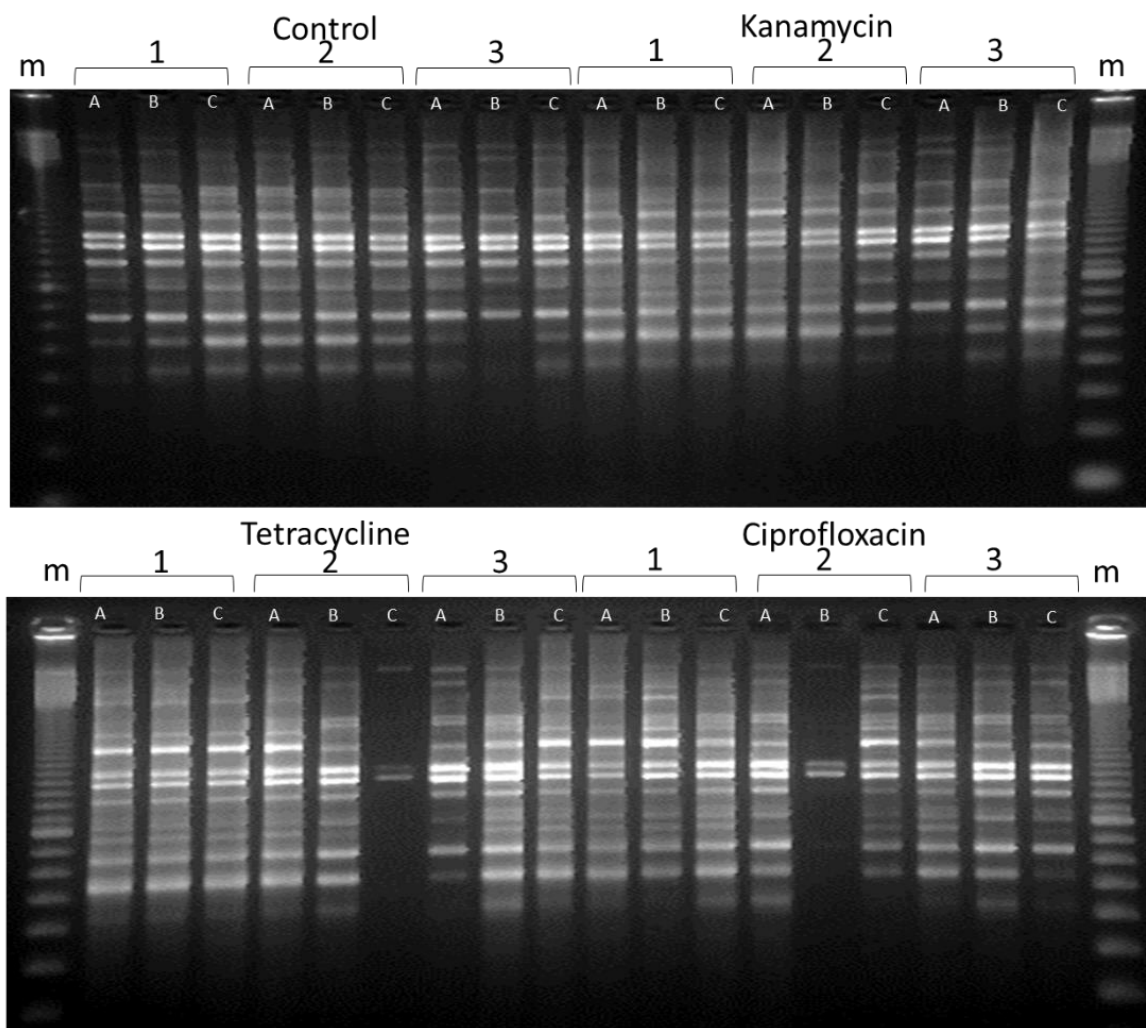

Supplement: Supplementary file 4 [file Image4.PDF]

Figure S4. BOX-PCR of generation 40 *Ps. aeruginosa* PA14. m = 100bp ladder

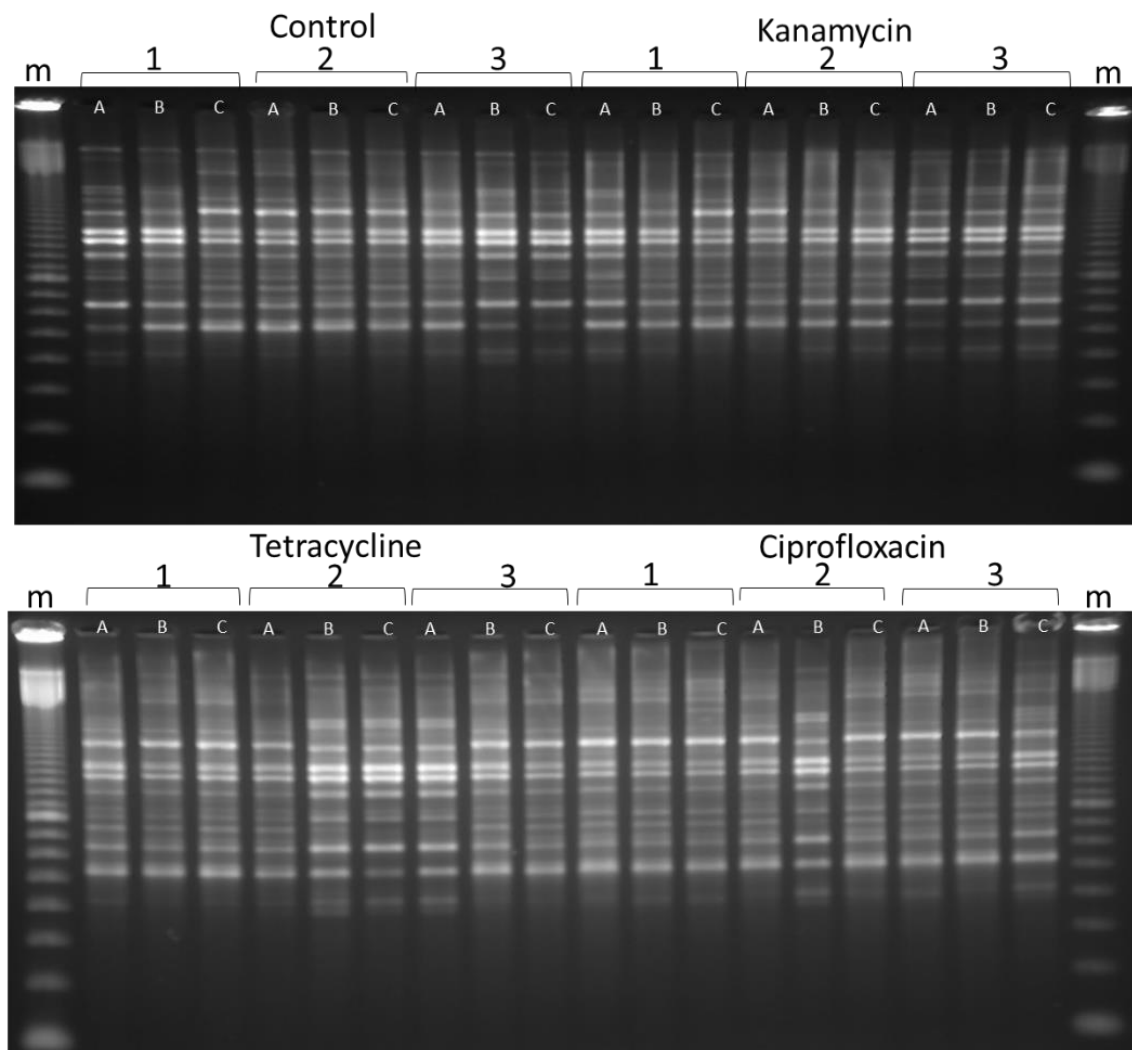

Supplement: Supplementary file 5 [file Image5.PDF]

**Figure S7.** MIC of antibiotics for the tested bacteria at generation 40

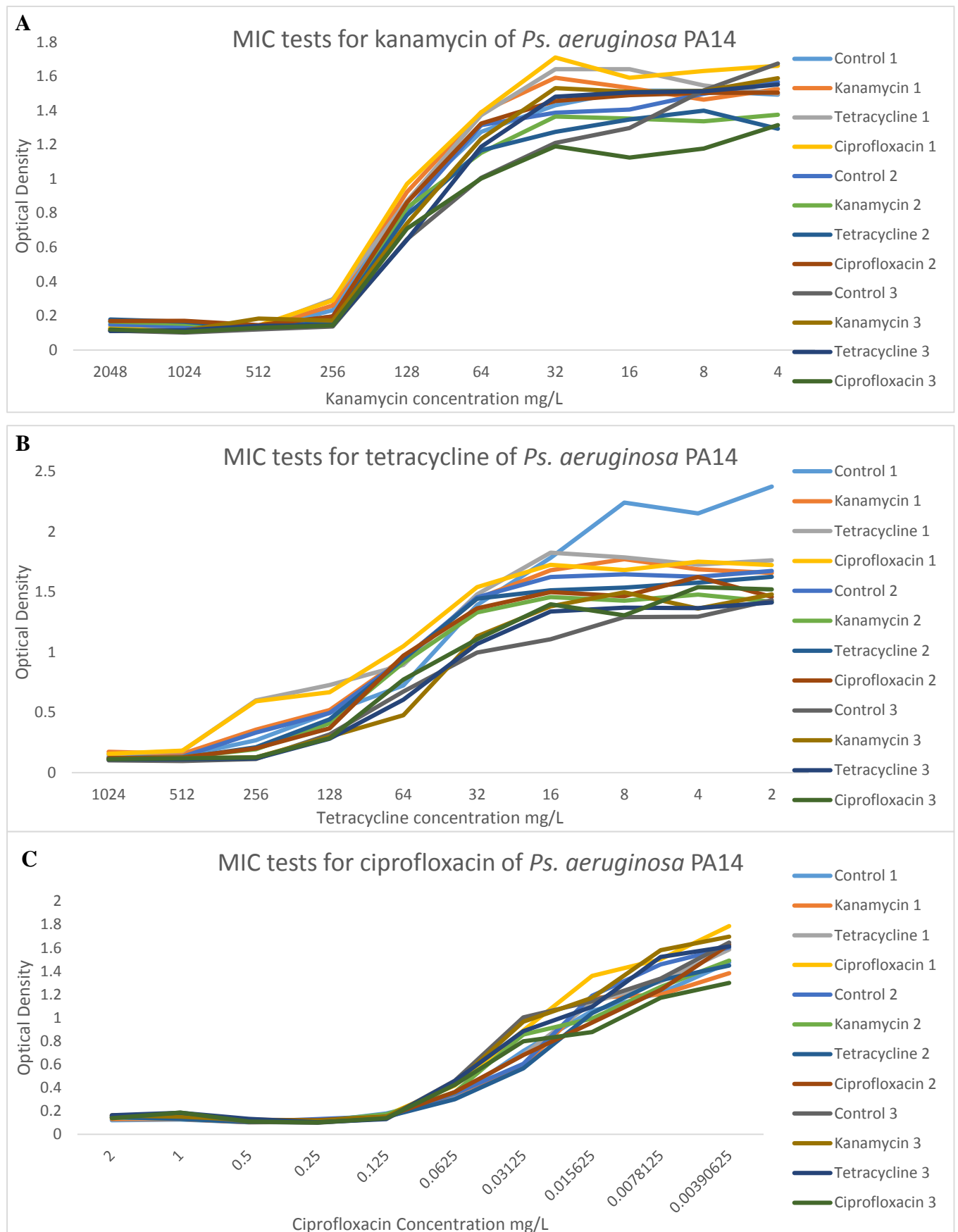

Supplement: Supplementary file 7 [file Image7.PDF]

**Figure S8.** MIC of antibiotics for the tested bacteria at generation 40

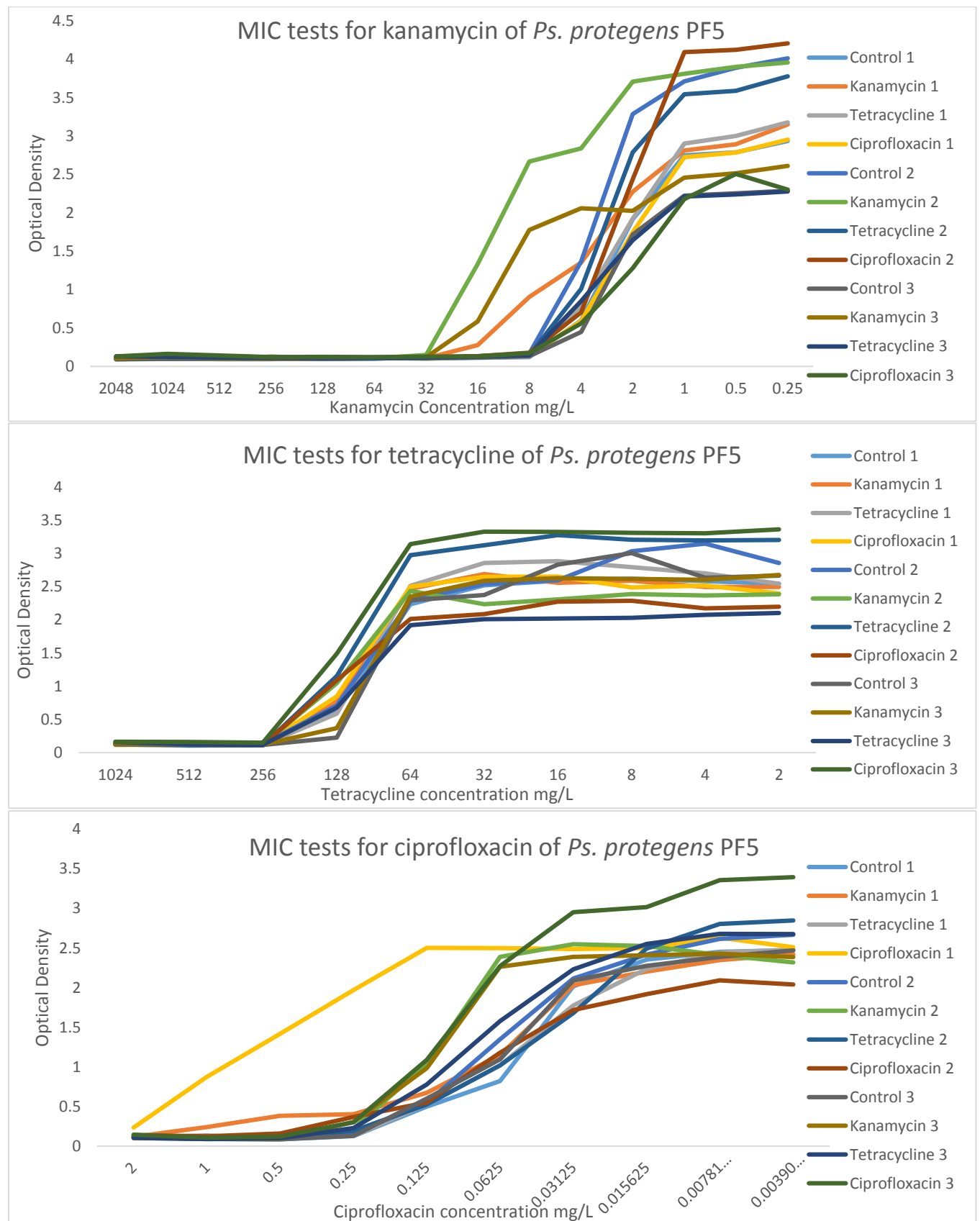

Supplement: Supplementary file 8 [file Image8.PDF]

**Figure S9.** MIC of antibiotics for the tested bacteria at generation 40

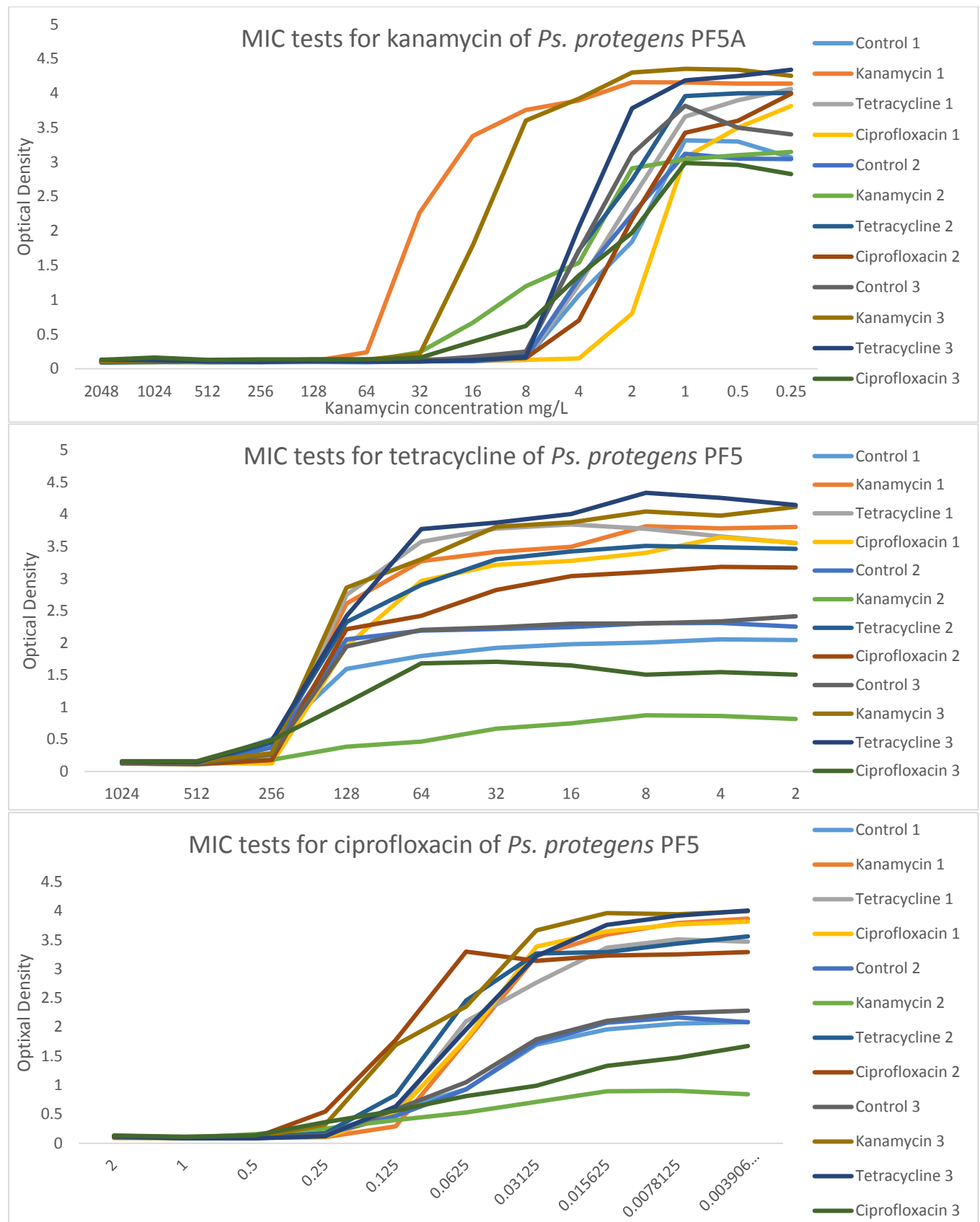

Supplement: Supplementary file 9 [file Image9.PDF]
