# Supplementary material for: Potential impacts of aquatic pollutants: sub-clinical antibiotic concentrations induce genome changes and promote antibiotic resistance
Source: Front Microbiol. 2015 Aug 5;6:803. doi: 10.3389/fmicb.2015.00803 (PMC4525061; doi:10.3389/fmicb.2015.00803)

Figure S2. *Pseudomonas protegens* PF-5A colony morphology at generation 40

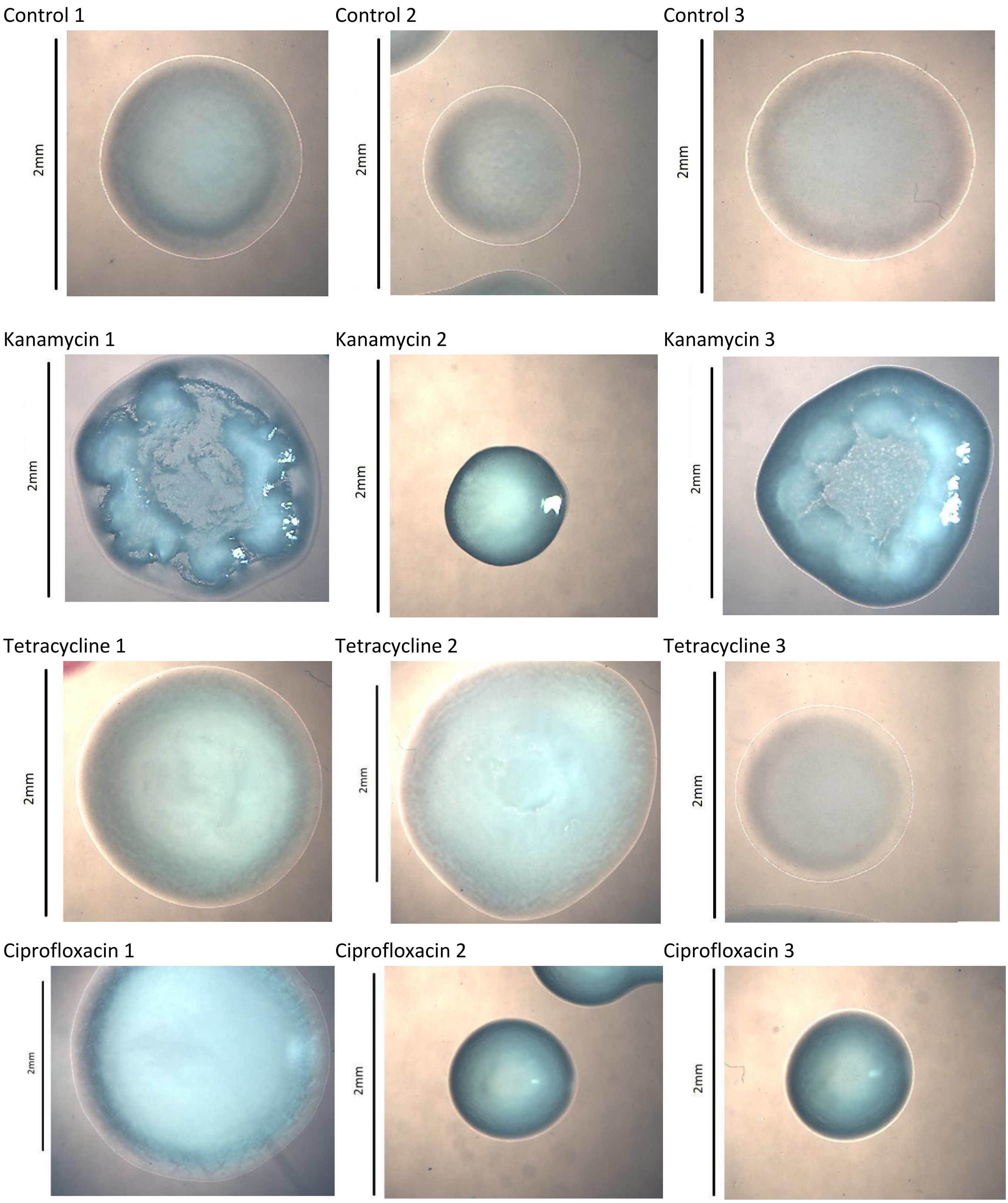

Supplement: Supplementary file 2 [file Image2.PDF]
