# Supplementary material for: Potential impacts of aquatic pollutants: sub-clinical antibiotic concentrations induce genome changes and promote antibiotic resistance
Source: Front Microbiol. 2015 Aug 5;6:803. doi: 10.3389/fmicb.2015.00803 (PMC4525061; doi:10.3389/fmicb.2015.00803)

Figure S6. BOX-PCR of generation 40 *Ps. protegens* PF-5A. Bands m are 100bp ladder. The band marked NG is a negative control with genereleaser. The band marked N is a negative control without genereleaser

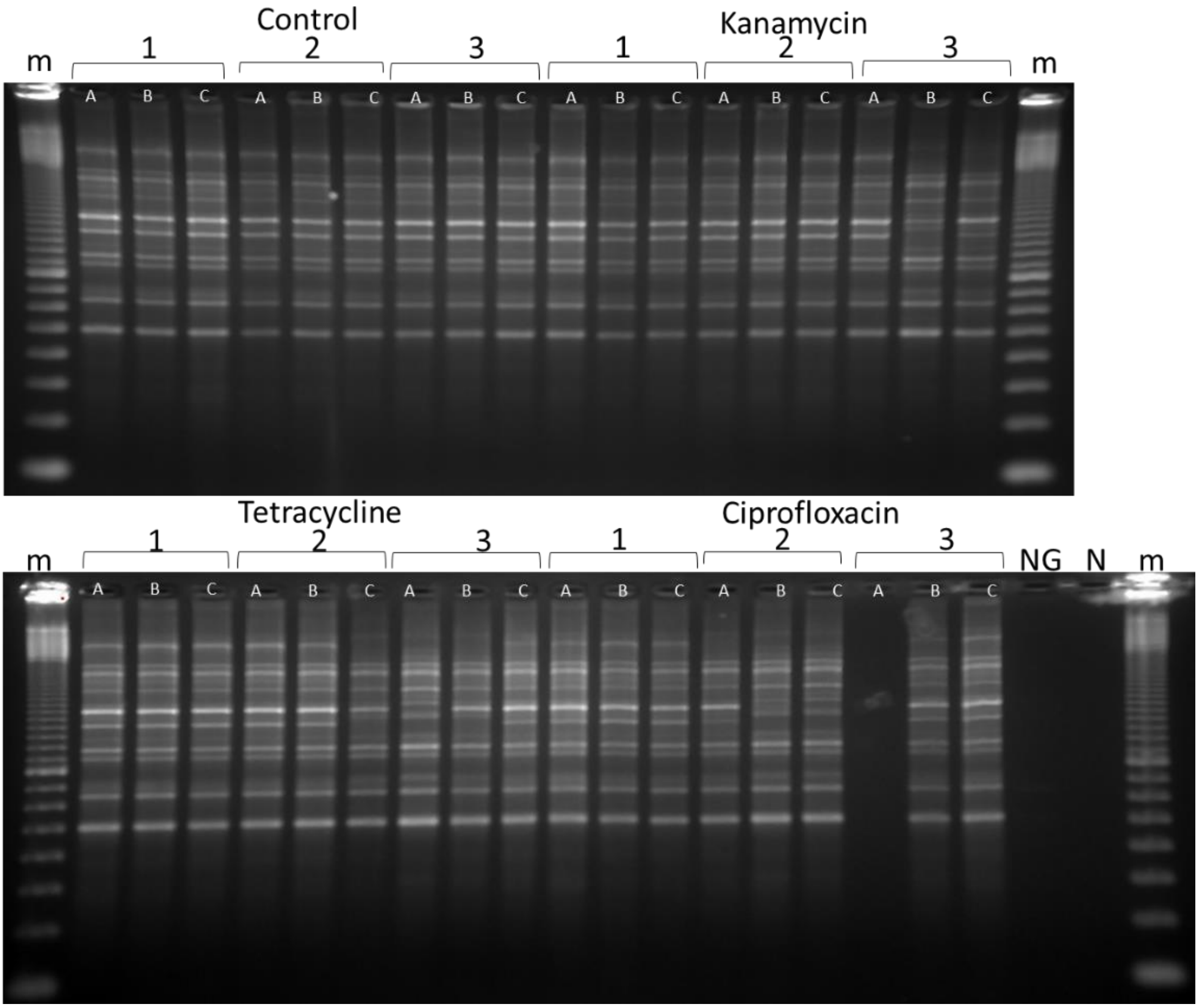

Supplement: Supplementary file 6 [file Image6.PDF]
